# Notes from Practice

**Published:** 1874-06-15

**Authors:** J. Schneck

**Affiliations:** Mt. Carmel, Illinois


THE
### MEDICAL EXAMINER.
A
*Semi-Monthly Journal of Medical Sciences.*

EDITED BY N. S. DAVIS, M.D., AND F. H. DAVIS, M.D.

No. XII. CHICAGO, JUNE 15, 1874. VOL. XV.

### Original Communications.

### NOTES FROM PRACTICE.

BY J. SCHNECK, M.D., MT. CARMEL, ILLINOIS.

ABOUT December 1st, 1872, the epizootic, which had been spreading over the eastern part of the United States and gradually wending its way westward, put in an appearance in our vicinity; and almost all horses, good and bad, were more or less severely affected with it.

In a few weeks after this onset, cases of erysipelas began to be frequently met with, (usually beginning on the face;) and by the first of February (two months), we found ourselves in the midst of an epidemic of erysipelas.

Simultaneously with the beginning of the erysipelas, many of our obstetric cases would have a "bad getting up"; and by February, cases of puerperal metro-peritonitis were met with, here and there. Soon after this, cases of cerebro-spinal meningitis were heard of, and seen, in almost all localities of our county and vicinity; in some localities producing fearful havoc, being usually fatal; but in our own immediate precinct, it never became very abundant or virulent.

During all this time, there were no cases of scarlatina or diphtheria heard of in our neighborhood. This condition of things continued, more or less violent, until about the first of April; when with the approach of pleasant weather the epizootic, erysipelas, puerperal metro-peritonitis and cerebro-spinal meningitis had all gradually disappeared; and no more was heard of either until this winter (1873-74), there having been a few sporadic cases of the latter disease. Of the erysipelas patients, there were no fatal cases, that I heard of, although, on a rough counting up, I find there were fully 300 cases treated by the physicians of this place. The sheet anchor in all cases was: Mur. tinct. iron and quinine. Of the spotted fever cases, I treated but three, all of which recovered; the treatment employed was large doses of bromide of potassium, and a blister to the nape of the neck for the first 12–20 hours; then quinine and stimulants, with fl. ex. calabar bean and ergot; and in two, where there were symptoms of effusion into the cavities of the brain, blisters to all four extremities were resorted to.

Of the cases of puerperal metro-peritonitis, all fully developed cases died; and in all cases the immediate cause appeared to be some injudiciousness on the part of the patient. But three cases occurred in my practice. The first was doing well until forty-eight hours after labor, when the disease was ushered in with a chill. I was called and, upon inquiry, found she had sat up in bed, talked and laughed a great deal during the first twenty-four hours, contrary to the most positive orders I had left in the morning; she died on the fifth day after accouchement, and third day of the disease. This was a large, rugged, but not very fleshy woman, aged thirty-four years; sixth accouchement; labor natural and easy.

The second was a large and fleshy person, in her fifteenth confinement, aged forty years. Had three convulsions before labor was terminated, and seven during the two days after, during most of which time she was very restless; the convulsions stopped after copious depletion and free use of diuretics. In this case, also, the disease was ushered in with a chill, on the fourth day after labor; and in five days more she was a corpse.

The third case was a lean, weakly person, in her first confinement; was doing well until the fifth day after labor, when her parents made her a visit; she sat up and talked with them, and made one trip to the kitchen; that evening metro-peritonitis was ushered in by a chill, and in four days she was dead.

The symptoms in the foregoing cases were very uniform; rigors at the outset: exceedingly rapid and irregular pulse; hurried respiration; suppression of milk and lochia, vomiting and diarrhœa; abdominal tenderness and swelling; tongue coated and dry; delirium and death. The treatment employed was somewhat varied; veratrum and opium, fomentations and poultices were mainly relied on.

Of the eighteen other cases of labor that I attended from December 1, 1872, to April 1 following, all had a moderately good recovery; but in all cases, I have good reasons to believe, my directions were fully carried out, *i. e.*, keep quiet, and in the recumbent position, and as free from all excitement as is possible. In addition to this, in most of the cases, an antiseptic was given, both before and during accouchement. Not being previously engaged in some cases, this rule was not strictly adhered to. The antiseptics used were sodæ sulphis or mur. tinct. iron, with bromochlorolum as a disinfectant. The conclusions that may be drawn from the foregoing imperfect history are:

(*a*) That, in the diseases above mentioned, including the epizootic, there is an origin from similar causes, viz.: Epidemic, (let the materies morbi be what it may.)

(*b*) Though scarlatina and diphtheria may appear, in some instances, to have acted the part of cause or effect to metro-peritonitis, in this instance no such cause or effect can be attributed to them.

(*c*) Our main reliance for saving the lives of parturient women during an epidemic of metro-peritonitis, is in prophylactic treatment.

(*d*) If antiseptics are the best prophylactics, why are they not the most curative?

P. S.—We should be glad to hear from other parts of the State on this subject.

